# An analytic model for Throughput Optimal Distributed Coordination Function (TO-DCF)

**DOI:** 10.1007/s11235-017-0275-6

**Published:** 2017-02-03

**Authors:** Emma Fitzgerald, Ulf Körner, Bjorn Landfeldt

**Affiliations:** Department of Electrical and Information Technology, Lund University, 221 00, Lund, Sweden

**Keywords:** Wireless LAN, MAC, 802.11, CSMA

## Abstract

TO-DCF, a new backoff scheme for 802.11, has the potential to significantly increase throughput in dense wireless LANs while also opportunistically favouring nodes with heavier traffic loads and/or better channel conditions. In this paper we present an analytical model to investigate the behaviour and performance of the TO-DCF protocol with regards to operating parameters such as the number of nodes, the contention window size and the backoff countdown probabilities. We then compare numerical results from an implementation of our model with simulations. Our model shows a high level of accuracy, even when the model assumptions are relaxed, and provides guidance for network operators to correctly configure the weight functions for nodes running TO-DCF given the network’s operating conditions.

## Introduction

Wireless LANs are moving towards denser, overlapping networks, with an increasingly demanding application mix as interactive and streaming-based applications become more and more popular. This calls for both more efficient protocols at the medium access control layer, and an increased focus on quality of service. A new backoff scheme for the IEEE 802.11 DCF, called Throughput-Optimal DCF (TO-DCF), was proposed in [16]. This scheme is able to opportunistically favour users with better channel conditions and higher traffic loads, and the results presented in [16] show significantly increased throughput over the standard DCF. In this work, we now provide in-depth analysis of this scheme as well as a numerical evaluation and comparison to simulations.

TO-DCF modifies the standard 802.11 DCF by introducing a countdown probability, unique to each node and dependent on its current transmission queue length and channel conditions. In the standard DCF, nodes decrement their backoff counters deterministically in every slot, whereas in TO-DCF this is done according to the countdown probability. These probabilities are assigned in such a way that nodes with higher traffic loads and better achievable transmission rates have higher probabilities to decrement their counters. Such nodes will thus statistically reach zero and attempt to transmit their head-of-line packets earlier.

As the work in [16] demonstrated, this mechanism has the potential to give large gains in performance, with the simulation results given showing improvements in throughput of up to 70% over standard 802.11. In addition, it has the advantage of simplicity and compatibility with the extensive userbase of existing 802.11 devices. However, in a practical deployment of this scheme, it is critical to have a thorough understanding of how the system behaves. This allows operators to choose appropriate values for the various parameters—in particular the countdown probabilities—depending on the current operating conditions and the goals of the network administrator.

In the current work, we analyse the TO-DCF backoff scheme with respect to various factors, such as the distribution of the countdown probabilities, the number of nodes in the network, the size of the contention window, and the nodes’ initial queue lengths. We also incorporate packet arrivals during backoff into the analysis, modelling the arrival process at each node with both a Poisson process and a process with arbitrarily high variance. TO-DCF has multiple, sometimes competing, goals, in particular the maximisation of overall throughput, the minimisation of delay, and improved QoS by favouring nodes with higher current traffic loads. We examine the trade-offs between these goals by deriving the following quantities: the expected backoff time, the probability that the node assigned the highest countdown probability is indeed the one that transmits first after backoff, the probability that this node is still the one with the highest traffic load by this time, and the collision probability. We created a numerical implementation of the model to evaluate these outputs and present concrete results for a range of different inputs. These are then compared to simulations of the system to examine the accuracy of the model as well as the system behaviour when the model assumptions are relaxed.

Our results show an important trade-off between the expected backoff time—that is, delay due to the backoff scheme—and the success of the protocol in terms of achieving the goals outlined above. In particular, we find that in order for the highest-weighted node to win contention and successfully transmit its packet first, the countdown probabilities need to be carefully tuned. Correctly-chosen countdown probabilities allow time for the highest-weighted node’s statistical advantage to have an effect, while not causing the nodes to spend an excessive time in backoff leading to unnecessary delays.

The rest of this paper is organised as follows. Section 2 examines related work in this area. Section 3 gives an overview of the TO-DCF backoff scheme and details our model for analysing its behaviour, and our analysis is then carried out in Sect. 4. Next, our simulations are described in Sect. 5 and our results are presented in Sect. 6. Finally, Sect. 7 concludes this paper.

## Related work

In Tassiulas and Ephremides [32] developed a centralised max-weight scheduling algorithm that is throughput-optimal. TO-DCF provides instead a distributed scheme that can obtain the same throughput optimality. There has been previous work aiming at distributed, throughput-optimal algorithms for CSMA systems, however, this work exhibits a number of drawbacks which TO-DCF addresses. Optimality for many of these algorithms [13, 23, 25, 27–29] only holds for non-fading channels. In [11], focus is placed on reducing the delay inherent in many CSMA-based approaches, however once again this is done using non-fading channels. In the analysis presented here, we do not include channel conditions but rather focus on the fundamental behaviour of TO-DCF and on the packet arrival process. Nonetheless, the TO-DCF scheme as presented in [16] includes channel conditions in the node weighting function, and thus applies even to fading channels.

The algorithms presented in [14, 19, 21] do consider fading channels, however they also present practical difficulties in implementation, especially when integrated into a system with devices running the standard 802.11 DCF. The protocol in [21] assumes continuous backoff times, whereas in 802.11 backoff occurs in discretised slots. The solutions in [14] and [19] are suited for the 802.11 DCF, however they require the estimation of global network statistics, which are not easy to measure accurately at an individual node. TO-DCF, in contrast, only requires local information. In addition, in these two schemes the contention window, as in 802.11, only takes discrete values, causing performance degradation due to quantisation. In TO-DCF, the countdown probabilities are chosen from a continuous range, allowing backoff time to be finely tuned to suit current operating conditions.

Other opportunistic and probabilistic channel access schemes have also been proposed in recent years. A variant of the TO-DCF scheme providing dynamic reservations was presented in [15], while in [10], token passing is used to improve the idle and collision times of the base 8021.11 DCF. History-Based Probabilistic Backoff [26], and a further refinement of it in [22], tailor the basic binary exponential backoff algorithm to better suit the needs of mobile ad-hoc networks. In [3], a probabilistic approach based on two-dimensional, discrete-time Markov chains is used to provide proportional fairness with regards to nodes’ traffic loads, without needing to solve difficult (non-linear and non-concave) optimisation problems. TO-DCF, by contrast, takes into account channel quality to achieve throughput-optimality, albeit possibly at the cost of fairness. The scheme proposed in [7] uses probabilistic polling to achieve high throughput. However, this work is focused on wireless sensor networks, rather than traditional wireless networks, and as such primarily considers energy consumption and energy harvesting rates as metrics rather than queue lengths and channel states as are used in TO-DCF. Similar approaches have also been proposed for new and emerging network types. The protocol presented in [4] brings a probabilistic approach to medium access control in full-duplex networks, and [30] describes opportunistic medium access control for cognitive radio sensor networks.

In our analysis in this paper, we work from first principles, beginning with the probabilities of basic events such as a node decrementing its backoff counter or the arrival of a packet within a given time. We then take sums and products over probability distributions in order to build up expressions for probabilities of more complex events. This kind of stochastic analysis has a long history of use in the domain of wireless networks, beginning with early analysis of ALOHA [1] and CSMA [18]. It has seen wide application in the area of stochastic geometry [9]. and work on wireless networks applying such an approach often arises from this [6, 8, 31]. However, it has also been used in other contexts, such as stochastic physical-layer channel models [12], and stochastic queueing networks [33].

An alternative approach to probabilistic analysis in networks is the use of Markov chains, as are frequently used in queueing theory [17] and were applied to the 802.11 DCF in [2]. However, in our case, Markov chain analysis becomes intractable due to the large number of different states and transitions. In TO-DCF, each node has its own specific countdown probability, resulting in the need for a separate Markov chain for each node. In addition, we wish to model packet arrivals, which makes a protocol stage-based Markov chain such as used in [2], where time is not directly modelled, unsuitable for the outcomes we wish to study.

## Analytic model

We will now give a summary of the TO-DCF backoff scheme, although readers are referred to [16] for further details. Following this, we will describe the model used for our analysis. This model focuses on a single instance of backoff in which all nodes begin the backoff process simultaneously, thus allowing us to directly compare their behaviour on an equal footing.

### The TO-DCF backoff scheme

Throughput-Optimal DCF (TO-DCF) is a variation on the standard 802.11 DCF in which nodes are given advantage during backoff according to a weight function. Each node computes the weight function independently. This function takes the node’s current queue length and channel quality as inputs, and uses them to compute a real number between 0 and 1 such that a longer queue or a better quality channel results in a higher output from the function. Accordingly, nodes that have a longer queue and/or more favourable channel conditions (and thus a higher datarate) receive a higher weight. (See [16] for further details on how the weight function is defined and computed.)

There are many different possible weight functions that fit the above criteria, and the exact function chosen will have an effect on the protocol’s performance. A function that does not provide a sufficient spread in weights for nodes with different queue lengths or channel conditions would result in a high number of collisions, while a too-large spread may result in unnecessary channel access delays and idle times. We do not directly consider the weight function in this work, however we analyse the effects of differing weight spreads by varying the actual backoff probabilities resulting from different weights, as described below.

The weights are used as follows. Each node chooses a backoff counter uniformly at random from the contention window as in standard 802.11. However, instead of deterministically decrementing this counter in every timeslot, in TO-DCF each node is assigned a countdown probability *p*. Nodes with higher weights are given higher countdown probabilities, and in each slot the backoff counter is decremented with probability *p*. For $p = 1.0$, then, TO-DCF is exactly equivalent to the 802.11 DCF.

The aim of this protocol is for nodes with higher weights to countdown more quickly and thus transmit sooner, with the ultimate goal being that the highest-weighted node, denoted $n^*$, counts down fastest and wins the channel. Since the different backoff probabilities provide only a statistical, rather than deterministic, advantage to higher-weighted nodes, it is entirely possible for a lower-weighted node to in fact win the channel in any given backoff period. Over time, however, higher-weighted nodes will have on average shorter backoff times and win contention more often. As with the 802.11 DCF, performance depends on the overall length of the backoff process—and thus the amount of time the channel spends idle before a node attempts transmission—as well as the collision probability. A collision occurs when two (or more) nodes attempt to transmit simultaneously, resulting in the failure of both (all) transmissions.

### Model assumptions

In our analysis, we consider a single backoff process, beginning when the channel becomes idle after a previous transmission, and ending once any node attempts transmission, whether or not a collision occurs. We also take some simplifying assumptions in order to make the analysis tractable. These are as follows.

1. *All nodes begin backoff simultaneously* In reality, a node may begin backoff at any time, as a packet may arrive causing it to join an ongoing backoff process, or it may have been unsuccessful in the previous process and thus have a residual backoff counter to count down. We however consider all nodes to begin backoff together and consider only a single backoff process, independent of previous events.
2. *The set of nodes is constant* No node joins or leaves the backoff process whilst it is underway.
3. *The countdown probabilities do not change during backoff* Although the countdown probabilities are a function of the node weights, we assume they are calculated once at the start of backoff and then remain constant until backoff ends.
4. *Nodes are synchronised* Timeslots begin and end at the same time for all nodes.
5. *The contention window is the same for all nodes* In reality the contention window will increase as a node experiences collisions, however we instead consider it to be constant for all nodes.

## Analysis

We wish to derive expressions for a number of quantities that will provides measures of the performance of the TO-DCF protocol. First, we are interested in the success of the protocol in causing the highest weight node to transmit first. However, it is possible that the parameters to the weight function, that is, the node’s channel conditions and queue length, change during the backoff process. Thus we also wish to determine how likely it is that the highest-weighted node at the beginning of the backoff process, $n^*$, still has the highest weight at the end. If not, it is no longer the most desirable node to transmit. In the following analysis, we do not consider channel conditions, only the queue length as it varies according to a packet arrival process.

Both of the above measures depend on the total duration of the backoff process, that is, the time from when backoff starts until any node attempts transmission, successfully or otherwise. We thus need to first derive an expression for the probability of backoff ending at a given time. Finally we will also derive expressions for the expected backoff time and for the probability that backoff ends with a collision.

### End of backoff time

*Aim* Find the probability that backoff ends at a given time *t*.

We will denote with *T* the time at which backoff ends, and we therefore wish to find $P(T = t)$, the probability that backoff ends at a given time *t*. This can only occur if no node attempts transmission before time *t*, and at least one node attempts transmission precisely at time *t*. Even if more than one node attempts transmission simultaneously, such that the transmission will result in a collision and fail, we nonetheless consider backoff to have ended. A new backoff process will then begin between any nodes that still have a packet to transmit.

Each node *n* has a backoff counter $b_n > 0, \ b_n \in \mathbb {Z}$. At each timeslot, $b_n$ has a probability $p_n$ to be decremented, and otherwise keeps its current value with probability $1 - p_n$. The countdown probability $p_n$ is determined by a function of the node’s weight $w_n$, with increasing weights giving increasing countdown probabilities, i.e. $w_n> w_m \implies p_n > p_m$. Here, we assume that $p_n$ is calculated once at the beginning of backoff and remains constant during the entire backoff process. This means that even if new packets arrive at node *n*, causing a change in $w_n$, $p_n$ will not change until backoff is finished as it is only based on the queue length when backoff began.

While it is entirely possible in a practical implementation to adjust the backoff probability *p* in case of changes in queue length during backoff, it significantly complicates the analysis and so we do not use this method. We do however compute the probability that node weights will change sufficiently during backoff, due to newly arriving packets, to affect which node has the highest weight. As will be shown in our numerical results, presented in Sect. 6, this probability remains low, even for highly variable packet arrival rates. This means that the assumption of constant backoff probabilities during each backoff period does not significantly affect the analysis of the protocol’s performance.

Let the probability that a node *n*’s backoff counter is decremented exactly *k* times in a period of *s* timeslots be denoted by $D_n(k, s)$, regardless of exactly when the counter is decremented. Then $D_n(k, s)$ is given by the binomial probability

1 $$\begin{aligned} D_n(k, s) = {s \atopwithdelims ()k} {p_n}^{k} (1 - p_n)^{(s - k)} \end{aligned}$$

Also, let the probability that node *n* will transmit at time *t* be denoted by $\tau _{n}^{c}(t)$, again, for *c* a given value of $b_n$. Then

2 $$\begin{aligned} \tau _n^{c}(t) = {\left\{ \begin{array}{ll} 0 &{} t < c \\ D_n(c - 1, t - 1)p_n &{} t \ge c \end{array}\right. } \end{aligned}$$

(Recall that $p_n$ is node *n*’s probability to decrement its backoff counter each timeslot.)

The first case indicates that the time is not long enough for the backoff counter to decrement to 0, even if the node were to decrement it in every timeslot. In the second case, the backoff counter must be decremented to 0 precisely as we reach timeslot *t*. For this to occur, the counter must be decremented in a total of *c* timeslots, and not decremented (held constant) in the remaining $c - t$ timeslots. The last decrement must occur in the last available timeslot, otherwise the node would have transmitted its packet earlier. If the counter has already been decremented $c-1$ times, then the probability for the last decrement to occur in the final slot is $p_n$. For the previous $c - 1$ decrements, however, it does not matter in which timeslots they occur, only the number of times the counter is decremented. Hence we have $D_n(c - 1, t - 1)$, with

3 $$\begin{aligned} D_n(c - 1, t - 1) = {t-1 \atopwithdelims ()c-1} {p_n}^{c-1} (1 - p_n)^{(t - c)} \end{aligned}$$

We can now sum over the distribution of the initial backoff counter values to find $\tau _{n}(t)$, the total probability that node *n* will transmit at time *t*.

4 $$\begin{aligned} \tau _{n}(t) = \sum _{c = 1}^{CW} \tau _{n}^{c}(t) P(b_n = c) \end{aligned}$$

The backoff counter $b_n$ has a uniform distribution over the interval [1, *CW*] and so we have

5 $$\begin{aligned} P(b_n = c) = \frac{1}{CW} \end{aligned}$$

for $$c \in [1, CW]$$.

The probability that node *n* does not transmit before time *t* is then given by

6 $$\begin{aligned} 1 - \sum _{i = 1}^{t-1} \tau _n(i) \end{aligned}$$

i.e. *n* does not transmit in any slot up until *t*. We will denote the probability that no node transmits before time *t*, that is, all nodes remain silent until that time, by *S*(*t*). Then we have

7 $$\begin{aligned} S(t) = \prod _{n \in \mathcal {N}} \left( 1 - \sum _{i = 1}^{t-1} \tau _n(i) \right) \end{aligned}$$

For backoff to end at time *t*, it is not enough for all nodes to remain silent during timeslots $0...t-1$. We require also that at least one node attempts transmission during slot *t*. Consider the probability that a node *n* attempts transmission at time *t*, given that it has not attempted transmission (has remained silent) up to and including time $t - 1$, i.e. $\tau _{n}(t)$ conditioned on Eq. 6. Let this probability be denoted $\chi _{n}(t)$, then

8 $$\begin{aligned} \chi _n(t)&= \frac{\tau _{n}(t)}{\sum _{i=t}^{\infty }\tau _{n}(i)} \nonumber \\&= \frac{\tau _{n}(t)}{1 - \sum _{i=1}^{t-1}\tau _{n}(i)} \end{aligned}$$

with the second form given being more computationally tractable since the sum is finite.

Here, $\chi _{n}(t)$ is essentially re-normalising $\tau _n(t)$ to cover only the remaining timeslots. We assume that the node has remained silent for all slots before time *t*, and hence all the probabilities $\tau _n(s), \ s < t$ must be removed from the distribution. It is no longer possible for a transmission to occur in these slots, and so our event space now only contains slots at or later than *t*.

To illustrate this, consider an example with $p_n=1.0$ (i.e. the node will count down unconditionally in every slot) and $CW=4$. Then we have $\tau _n(t) = 0.25$ for $1 \le t \le 4$ and $\tau _n(t) = 0$ otherwise. The transmission time will depend only on the initial choice of the backoff counter $b_n$. Now we wish to find $\chi _n(t)$. For $t=1$, we have the same value as for $\tau (1)$ (0.25), as there have been no preceding silent slots. However, for $t=2$, we now take the condition that the node did *not* transmit in slot 1. We then have three remaining slots, each with equal probability, and so $\chi _n(2) = \frac{1}{3}$. Similarly, $\chi _n(3) = 0.5$ and $\chi _n(4) = 1.0$ — if the node has not transmitted in any of slots 1–3, it *must* then transmit in slot 4.

Then the probability that at least one node transmits at time *t*, given that no node transmits before then, is given by

9 $$\begin{aligned} 1 - \prod _{n \in \mathcal {N}} \left( 1 - \chi _n(t) \right) \end{aligned}$$

Finally, the probability that backoff ends at time *t* is given by

10 $$\begin{aligned} P(T = t) = S(t)\left( 1 - \prod _{n \in \mathcal {N}} \left( 1 - \chi _n(t) \right) \right) \end{aligned}$$

$$\square $$

### $n^*$ transmits first

#### Successful transmission

*Aim* Find the probability that node $n^*$ (i.e. the node with highest weight at the beginning of the backoff process) transmits first and does so successfully, that is, without collision.

For $n^*$ to transmit first, it must transmit at time *T*, that is, when backoff ends. For this transmission to also be successful, no other node may transmit at this time. We can derive the probability of this occurring from Eqs. 8 and 9. For a given *T*, the probability that $n^*$ sends first without collision is

11 $$\begin{aligned} \chi _{n^*}(T)\left( \prod _{n \in \mathcal {N}, n \ne n^*}\left( 1 - \chi _{n}(T)\right) \right) \end{aligned}$$

We can then sum over the distribution of *T*, taking for each value of *T* the condition that all nodes are silent up until that point so that backoff will indeed end at that *T*. This then gives the total probability that $n^*$ sends first without collision, regardless of when backoff ends.

12 $$\begin{aligned} \sum _{t=1}^\infty \left( S(t) \chi _{n^*}(t)\prod _{n \in \mathcal {N}, n \ne n^*}\left( 1 - \chi _{n}(t)\right) \right) \end{aligned}$$

Note that $P(T = t)$ was already derived in Eq. 10. $\square$

#### Transmission regardless of collision

*Aim* Find the probability that node $n^*$ transmits first, regardless of the success of this transmission.

We can also find the probability that $n^*$ transmits first, regardless of whether or not other nodes also transmit at the same time. This gives a measure of the success of the assignment of countdown probabilities to nodes, setting aside the overall success of the backoff process and resulting transmission. To find this probability, we remove the product over the set of nodes from Eq. 12. This then removes the condition that other nodes must remain silent during $n^*$’s transmission. Thus the overall probability that $n^*$ will transmit (possibly equal) first, is given by

13 $$\begin{aligned} \sum _{t=1}^\infty S(t) \chi _{n^*}(t) \end{aligned}$$

$$\square $$

### $n^*$ is still the highest-weighted node at the end of backoff

*Aim* Find the probability that $n^*$ will have the highest weight at the end of the backoff process.

The weight for each node depends on the node’s queue length. At the start of the backoff process, each node *n* calculates its weight $w_n(Q_n)$, where $Q_n$ denotes the length of *n*’s queue when backoff begins, i.e. at time $t=0$. The node $n^*$ has by definition the highest weight at the beginning of the backoff process. We therefore have $w_{n^*} \ge w_n \ \forall \, n \ne n^*$. The weight $w_n$ is a strictly monotonically increasing function of queue length, hence we also have $Q_{n^*} \ge Q_n \ \forall \, n \ne n^*$. Note that if there is more than one node with the longest queue then $n^*$ can be chosen amongst these nodes arbitrarily.

In order for some other node $n \ne n^*$ to “overtake” $n^*$ and become the new highest-weight node by the time backoff ends, *n* must have a longer queue than $n^*$ at that time. That is, the number of packet arrivals at *n* must be more than the original difference in queue lengths between *n* and $n^*$ plus any arrivals that have occurred at $n^*$ in the meantime, i.e.:

14 $$\begin{aligned} Q_{n} + A_{n} > Q_{n^*} + A_{n^*} \end{aligned}$$

where $A_i$ denotes the number of packet arrivals at node *i* during backoff.

Rearranging, we obtain

15 $$\begin{aligned} A_{n} > Q_{n^*} - Q_{n} + A_{n^*} \end{aligned}$$

Note that $Q_{n^*} - Q_{n}$ here is a constant as $Q_i$ denotes the length of node *i*’s queue at the beginning of backoff, whereas both $A_{n}$ and $A_{n^*}$ will depend on the duration of the backoff process.

Backoff ends when any node attempts transmission of its head-of-line packet. Let the timeslot in which this occurs be *T*, and the time at which backoff begins be $t = 0$. Since backoff occurs in discrete timeslots, we normalise time to the length of a timeslot. Then *T* must be a positive integer, and under TO-DCF *T* has no upper bound—it is possible, if unlikely, for no node to ever decrement its backoff counter.

16 $$\begin{aligned}&P(A_{n} - A_{n^*} > Q_{n^*} - Q_{n} | T = t) \nonumber \\&\quad = \sum _{j=0}^\infty \left( 1 - \prod _{\begin{array}{c} n\in \mathcal N,\\ n \ne n^* \end{array}} \left( 1 - \sum _{k=\hat{A}}^\infty P(A_n = k | T = t) \right) \right) P(A_{n^*} = j) \end{aligned}$$

17 $$\begin{aligned}&P(X = k)\nonumber \\&\quad = \alpha \frac{((1 - \alpha )\lambda t)^k}{k!} e^{-(1 - \alpha )\lambda t} + (1 - \alpha ) \frac{(\alpha \lambda t)^k}{k!} e^{- \alpha \lambda t} \end{aligned}$$

Then, the probability $o_n$ that node *n* will overtake $n^*$ to have a higher weight by the end of backoff is given by:

18 $$\begin{aligned} o_{n} = \sum _{t=1}^\infty P(A_{n} - A_{n^*} > Q_{n^*} - Q_{n} | T = t)P(T = t) \end{aligned}$$

To find the probability that $n^*$ is the node with the highest weight at the end of the backoff process, we must consider all other nodes. We want the probability that no other node will have overtaken $n^*$ at the end of backoff. Note that it is in fact possible for another node to temporarily overtake $n^*$, however, we are only concerned with the state of the nodes at time *T*, as the overall goal of the TO-DCF scheme is for the highest-weighted node to attempt transmission first.

It is possible for more than one node, or any combination of multiple nodes, to overtake $n^*$, by the end of backoff, so we instead take the probability that each node does *not* overtake $n^*$, given by $1 - o_n$. Hence the probability that $n^*$ is the highest-weighted node at the end of the backoff process is given by

19 $$\begin{aligned} \prod _{n \in \mathcal {N}, n \ne n^*} \left( 1 - o_n \right) \end{aligned}$$

where $\mathcal {N}$ is the set of all nodes performing backoff. Note that even if $n^*$ is indeed still the highest-weighted node when backoff ends, this does not imply that it was $n^*$’s transmission that ended the backoff process. It is entirely possible that another node’s counter reached zero before $n^*$’s. For full success in the protocol’s goal we require both that $n^*$ is still the highest-weighted node and that it transmits first. The probability that $n*$ transmits first is derived in Sect. 4.2

We now wish to find an expression for $o_n$. $P(T = t)$ is already given by Eq. 10. To find an expression for $P(A_{n} - A_{n^*} > Q_{n^*} - Q_{n} | T = t)$, we need to know the packet arrival process for each node. As an example, suppose packets arrive at each node *n* according to a Poisson process with parameter $\lambda _n$, and that these processes are independent. That is, the number of packets that will arrive at node *n* during backoff is

20 $$\begin{aligned} P(A_n = k | T = t) = \frac{e^{-\lambda _n t} \left( \lambda _n t\right) ^k}{k!} \end{aligned}$$

Suppose $n^*$ receives *j* arrivals during backoff. Then the probability of a node *n* overtaking $n^*$, that is, having a longer queue than $n^*$ at the end of backoff, is

21 $$\begin{aligned} \sum _{k=\hat{A}}^\infty P(A_n = k | T = t) \end{aligned}$$

where $\hat{A}=Q_{n*}-Q_n+j+1$. For at least one other node to overtake $n^*$, the probability is thus

22 $$\begin{aligned} 1 - \prod _{\begin{array}{c} n\in \mathcal N,\\ n \ne n^* \end{array}} \left( 1 - \sum _{k=\hat{A}}^\infty P(A_n = k | T = t) \right) \end{aligned}$$

and to obtain the unconditional probability that at least one node overtakes $n^*$, we then sum over the number of packet arrivals at $n^*$ during backoff (Eq. 16).

Here $n^*$’s arrival process is also Poissonian, so we have

23 $$\begin{aligned} P(A_{n^*} = j) = \frac{e^{-\lambda _{n^*} t} \left( \lambda _{n^*} t\right) ^j}{j!} \end{aligned}$$

$$\square $$

#### High variance arrival process

Real internet traffic displays high variance and self-similarity [5, 20, 24] and thus cannot be accurately modelled with a Poisson arrival process. Models for self-similar packet arrivals are more complex and difficult to analyse. However, in order to provide some insight into the behaviour of the TO-DCF scheme under conditions of high variance in packet interarrival times, we have developed for our analysis an arrival process that allows for arbitrarily high (albeit finite) variance for a given mean arrival rate.

24 $$\begin{aligned} E[X(X-1)]&= \sum _{k=0}^\infty k(k-1) P(X = k) \nonumber \\&= \sum _{k=0}^\infty k(k-1) \left( \alpha \frac{((1 - \alpha )\lambda t)^k}{k!} e^{-(1 - \alpha )\lambda t} \right. \nonumber \\&\quad \left. + \,(1 - \alpha ) \frac{(\alpha \lambda t)^k}{k!} e^{ \alpha \lambda t} \right) \nonumber \\&= \alpha ((1 - \alpha ) \lambda t)^2 e^{-(1 - \alpha )\lambda t}\sum _{k=2}^\infty \frac{((1 - \alpha )\lambda t)^{k-2}}{(k-2)!}\nonumber \\&\quad +\,(1 - \alpha )(\alpha \lambda t)^2 e^{- \alpha \lambda t}\sum _{k=2}^\infty \frac{(\alpha \lambda t)^{k-2}}{(k-2)!} \nonumber \\&= \alpha ((1 - \alpha ) \lambda t)^2 e^{-(1 - \alpha )\lambda t}\sum _{k=0}^\infty \frac{((1 - \alpha )\lambda t)^k}{k!} \nonumber \\&\quad + \,(1 - \alpha )(\alpha \lambda t)^2 e^{- \alpha \lambda t}\sum _{k=0}^\infty \frac{(\alpha \lambda t)^k}{k!} \nonumber \\&= \alpha ((1 - \alpha )\lambda t)^2 + (1 - \alpha )(\alpha \lambda t)^2\nonumber \\&= \alpha (1 - \alpha ) (\lambda t)^2)((1 - \alpha ) + \alpha ) \nonumber \\&= \alpha (1 - \alpha ) (\lambda t)^2 \end{aligned}$$

25 $$\begin{aligned} \mathrm {Var}(X)&= E[X(X-1)] + E[X] - (E[X])^2 \nonumber \\&= \alpha (1 - \alpha ) (\lambda t)^2 + 2 \alpha (1 - \alpha ) \lambda t - (2 \alpha (1 - \alpha ) \lambda t)^2 \nonumber \\&= \alpha (1 - \alpha )\lambda t(\lambda t + 2 - 4\alpha (1 - \alpha ) \lambda t) \nonumber \\ \end{aligned}$$

*Probability distribution function* The high variance arrival process is similar in concept to a Markov-modulated Poisson process. Its probability density function is given by Eq. 17.

This can be thought of as forming a Poisson process with mean arrival rate $\alpha \lambda$, with probability $\alpha$, and a Poisson process with mean arrival rate $(1 - \alpha ) \lambda$ with probability $(1 - \alpha )$. Here, $0 \le \alpha \le 1$. Thus, for $\alpha = 0.5$, it devolves to a Poisson process with mean rate $0.5\alpha \lambda$, however for small or large $\alpha$ (it is symmetric about $\alpha = 0.5$), the difference between the two terms in the probability distribution function increases.

*Mean* Equation 17 can be viewed as the sum of two Poisson processes, one with mean arrival rate $(1 - \alpha ) \lambda$ and scaled by $\alpha$, and the other with mean arrival rate $\alpha \lambda$ and scaled by $(1 - \alpha )$. Hence the mean arrival rate for the entire process is given by the sum of the arrival rates, that is

26 $$\begin{aligned} E[X]&= \alpha (1 - \alpha ) \lambda t + (1 - \alpha ) \alpha \lambda t \nonumber \\&= 2 \alpha (1 - \alpha ) \lambda t \end{aligned}$$

If a given mean arrival rate $\mu$ is desired, $\lambda$ can then be found as a function of $\mu$ and $\alpha$ as

27 $$\begin{aligned} \lambda = \frac{\mu }{2 \alpha (1 - \alpha )} \end{aligned}$$

It is thus possible to keep the mean arrival rate constant while varying $\alpha$ by adjusting $\lambda$ to compensate.

*Variance* The variance of the process is given by

$$\begin{aligned} \mathrm {Var}(X)&= E[X^2] - (E[X])^2 \\&= E[X^2] - E[X] + E[X] - (E[X])^2 \\&= E[X^2 - X] + E[X] - (E[X])^2 \\&= E[X(X-1)] + E[X] - (E[X])^2 \end{aligned}$$

We can derive $E[X(X-1)]$ from Eq. 17 as shown in Eq. 24. From Eqs. 26 and 24 we can then derive the variance (Eq. 25).

28 $$\begin{aligned}&P(\mathrm {success}) \nonumber \\&\quad = \sum _{t=1}^\infty \sum _{n \in \mathcal {N}} \left( S(t)\chi _{n}(t)\left( 1 - \prod _{m \in \mathcal {N}, m \ne n}\left( 1 - \chi _{m}(t)\right) \right) \right) \nonumber \\ \end{aligned}$$

*Index of dispersion* The index of dispersion of a probability distribution gives a measure of the variance normalised to the mean and is defined as

$$\begin{aligned} D(X) = \frac{\mathrm {Var}(X)}{E[X]} \end{aligned}$$

For our distribution, then, we have

29 $$\begin{aligned} D(X)&= \frac{\alpha (1 - \alpha )\lambda t(\lambda t + 2 - 4\alpha (1 - \alpha ) \lambda t)}{2 \alpha (1 - \alpha ) \lambda t} \nonumber \\&= 0.5(\lambda t + 2 - 4\alpha (1 - \alpha ) \lambda t) \nonumber \\&= 0.5\lambda t (1 - 4 \alpha +4 \alpha ^2) + 1 \nonumber \\&= 0.5\lambda t(2\alpha -1)^2 + 1 \end{aligned}$$

Since the first term is squared and thus always non-negative, Eq. 29 is always at least 1, which is the index of dispersion of a Poisson process. $D(X) = 1$ when $\alpha = 0.5$, in which case our distribution is identical to a Poisson distribution.

**Fig. 1:**
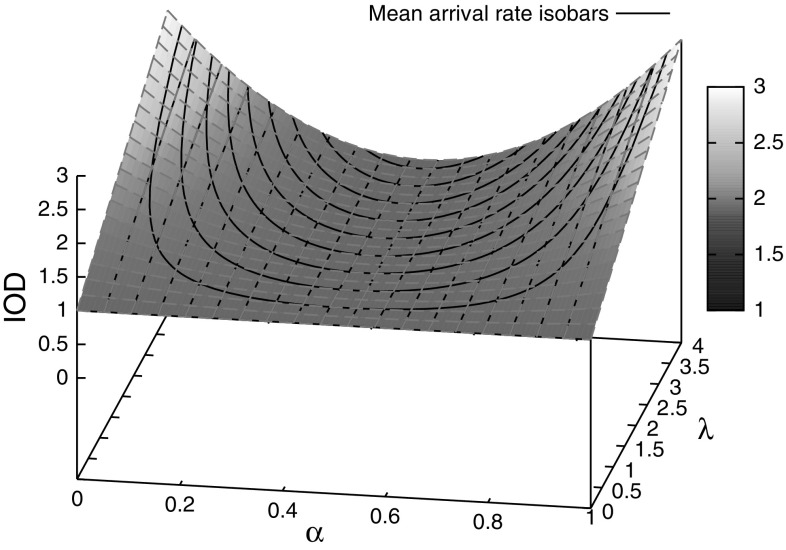
Index of dispersion for our packet arrival distribution

Moreover, the index of dispersion can be made arbitrarily high. For a given desired mean $\mu$, then we can see from Eq. 27 that $\lambda$ increases as $\alpha$ approaches either 0 or 1, and from Eq. 29 we see that the index of dispersion also grows with $\lambda$ as $\alpha$ approaches 0 or 1. Thus by taking $\alpha$ sufficiently close to 0 or 1 we can make the index of dispersion of the distribution arbitrarily high while keeping the same mean. Figure 1 shows the index of dispersion for the distribution for varying $\alpha$ and $\lambda$, with the black lines in the figure indicating mean arrival rate isobars, that is, curves along which the distribution has constant mean.

### Expected backoff time

*Aim* Find the expected duration of the backoff process.

The expected length of time before any node sends, that is, the amount of time the channel spends idle before a transmission is attempted, can be derived directly from the distribution of $P(T = t)$. It is given by

30 $$\begin{aligned} E[T] = \sum _{t=1}^{\infty } tP(T = t) \end{aligned}$$

$$\square $$

### Collision probability

*Aim* Find the probability that backoff ends in collision.

We first find the probability that backoff ends in a successful transmission without any collision. We can take the expression in Eq. 12 and adapt it to allow any node to transmit, not just $n^*$. To do this, we sum over all the nodes for each possible backoff duration (Eq. 28).

The collision probability is then given by the complement, $1 - P(\mathrm {success})$. $\square$

### Numerical implementation

We created an implementation of the analytical model using Python and Numpy, and calculated numeric results for a range of inputs. The outputs calculated were the probability that $n^*$ is still the highest weighted node at the end of backoff [denoted in the figures as $P(n^*\ \mathrm {remains\ highest\ weighted} \mathrm{node})$], the probability that $n^*$ will transmit first and without collisions [denoted in the figures as $P(n^*$sends first without collision)], the probability that $n^*$ will transmit first regardless of collisions [denoted in the figures as $P(n^*\ \mathrm {sends\ first})$], and the expected backoff time. These results were then compared with simulations as described in Sect. 5.

## Simulation

In order to test the correctness of our analytic model and to explore the effects of the assumptions made in Sect. 3.2, we created a discrete-time simulation in Python. The simulation contains a configurable number of nodes, each of which follow the TO-DCF protocol. At the beginning of the simulation, one node is chosen to be the initial $n^*$ node and the nodes are configured according to the input parameters. These include initial queue length, countdown probability, contention window, arrival rate, and $\alpha$ (see Sect. 4.3.1). These parameters are used to initialise the nodes’ queues and backoff counters.

Algorithm 1 shows the process for running the simulation. Time in the simulation progresses in slots, and in each slot, each node first decrements its backoff timer probabilistically according to the protocol. If the backoff counter has reached 0 in this timeslot, the node is added to a global list of transmitters (cleared at the beginning of each timeslot).

Once the nodes have executed these actions for the timeslot, the transmitter list is checked to see if there are any transmitters. If so, packets then arrive at each node according to the distribution given in Sect. 4.3.1. Since this distribution is intended to model bursty traffic, this is done by adding packets according to a Poisson distribution, with parameter $(1 - \alpha ) \lambda t$ with probability $\alpha$, and with parameter $\alpha \lambda t$ with probability $(1 - \alpha )$, where *t* is the time elapsed during backoff. We then record whether the node $n^*$ was among the transmitters, and if there was a collision, i.e. more than one transmitter. We also check whether $n^*$ still has the (equal) longest queue among all the nodes. These results are then output, along with the backoff time, that is, the number of slots until a node transmitted.
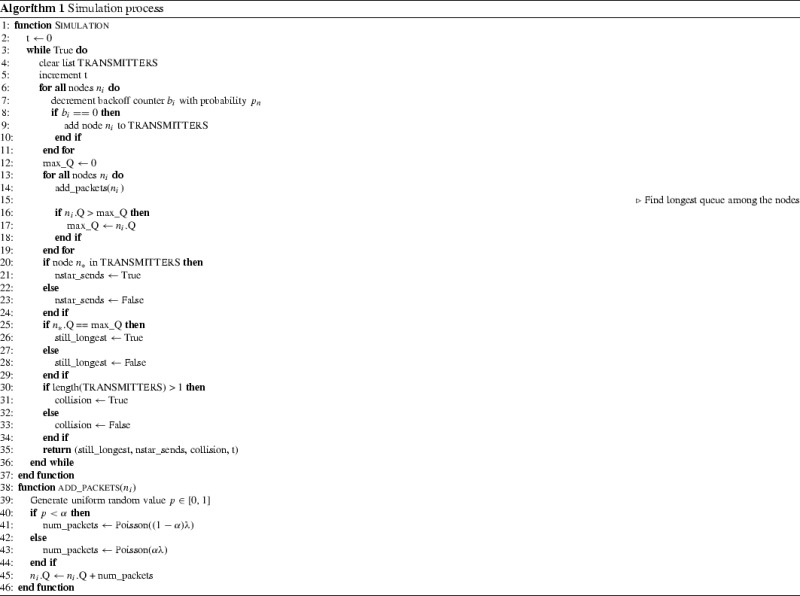


Initially, the simulation was configured to follow all the assumptions given in Sect. 3.2, however we then relaxed some of these assumptions for later experiments. In particular, the simulation included multiple backoff periods and exponential backoff. In the case of multiple backoff periods, $n^*$ was set to the node with the longest queue after each transmission, and the countdown probabilities of the nodes adjusted accordingly. Note that since packets are added to each node’s queue at the end of each backoff period, during each period, each node is either in a “burst” state, with high packet arrival rate, or a non-burst state with low packet arrival rate. We chose to adjust the arrival rate each backoff period rather than each slot as this more closely models bursts of traffic. In reality, the length of traffic bursts is application-dependent, however here we do not seek to provide completely accurate traffic modelling for a specific scenario, but rather give insight into the effect of bursty traffic on the performance of TO-DCF.

**Table 1:** Inputs

Input	Meaning	Values
*N*	Number of nodes, including $n^*$	2, 5, 10, 20
$Q_n$	Initial queue length for nodes other than $n^*$	1
$Q_{n^*}$	Initial queue length for $n^*$	2, 5, 10
$\lambda _n$	Packet arrival rate for nodes other than $n^*$	0.001, 0.005$^\mathrm{a}$
$\lambda _{n^*}$	Packet arrival rate for $n^*$	0.001, 0.005$^\mathrm{a}$
$p_n$	Per-slot countdown probability for nodes other than $n^*$	0.1...0.9, step 0.1$^\mathrm{b}$
$p_{n^*}$	Per-slot countdown probability for $n^*$	0.1...1.0, step 0.1$^\mathrm{b}$
*CW*	Contention window size	1, 4, 16, 32, 64
$\alpha$	Variance parameter for packet arrivals	0.0001, 0.01, 0.5
*BP*	Number of backoff periods$^\mathrm{c}$	1, 2, 5, 10, 20

$^\mathrm{a}$ The pair $(\lambda _n, \lambda {_n^*}) = (0.005, 0.005)$ was not included in the dataset

$^\mathrm{b}$ Only cases where $p_{n^*} \ge p_n$ were included

$^\mathrm{c}$ For multiple backoff period simulations only

## Results

We ran both the numeric model implementation and the simulation with the input values shown in Table 1. These values were chosen so as to give a range of operating conditions for the protocol and to investigate the effects of differing initial queue lengths, packet arrival rates, countdown probabilities, contention windows and packet arrival variance. All nodes other than $n^*$ received the same parameters, so that the set of all nodes $\mathcal {N}$ is partitioned into $n^*$ and $N-1$ identical nodes. The nodes other than $n^*$ were always given an initial queue length of 1, since in the analysis above, there is no dependence on this value directly but rather only on the difference $Q_{n^*}-Q_n$. Input values were chosen to be representative of realistic networks. In particular, the arrival rate values were chosen such that the network would not be saturated, that is, there should be on average less than one packet arrival per backoff period. Simulations for each set of input parameters were run 1000 times and 95% confidence intervals are shown in the figures presented in the following sections.

Four outputs were recorded for both the numeric implementation of the model and the simulations. These were

1. $P(n^*\,\mathrm {remains}\, n^*)$: the probability that $n^*$, the node that had the longest initial queue, also had the (possibly equal) longest queue when backoff ended by at least one node transmitting its packet
2. $P(n^*\,\mathrm {sends\,first\,without\,collision})$: the probability that $n^*$ transmitted its packet at the end of backoff, and was the only node to do so, i.e. no collision occurred
3. $P(n^*\,\mathrm {sends\,first})$: the probability that $n^*$ transmitted its packet at the end of backoff, regardless of whether or not a collision occurred
4. Backoff time: the number of timeslots until backoff was ended by a node attempting transmission

For the simulations, outputs 1–3 were proportions of the simulation runs in which the specified event occurred, rather than probabilities. For the numeric model implementation, output 4 was the expected backoff time, whereas for the simulations it was the average backoff time taken across all simulation runs.

### Model accuracy

The results of the numerical implementation of our model and the simulations agreed well. The relative error for each point in our dataset was calculated as

$$\begin{aligned} \mathrm {RE} = \frac{|S - M|}{M} \end{aligned}$$

where *S* indicates the simulation result and *M* indicates the model result. The average relative error across the entire dataset was 0.024.

75.2% of the model results generated from our dataset fell within the confidence interval of the corresponding simulation result. However, in many cases this is misleading as some input parameters generate very small or very large probabilities as outputs, so that the unlikely event may not occur even once in the entire set of 1000 simulation runs. To counter this, we calculated the percentage of model results that either fell within the confidence interval of the corresponding simulation result, or were within 0.05 (absolute error) of the simulation results, This gave a value of 92.2%.

### Uncertainty coefficients

In order to investigate the relative impact of the different input parameters, we calculated uncertainty coefficients for each parameter. The uncertainty coefficient for a random variable *X* given another random variable *Y* is defined as

$$\begin{aligned} U(X|Y) = \frac{I(X;Y)}{H(X)} \end{aligned}$$

That is, the uncertainty coefficient *U*(*X*|*Y*) is the mutual information of *X* and *Y* normalised to the entropy of *X*. Intuitively, the uncertainty coefficient gives a measure of how much of the variation in the variable *X* is explained by the variation in the variable *Y*. Note that unlike mutual information, *U*(*X*|*Y*) is asymmetrical, that is, $U(X|Y) \ne U(Y|X)$.

Here, we take our model results as the *X* variable, and our *Y* variable as the input parameters given in Table 1. This then tells us how much influence each parameter has on the model results.

**Fig. 2:**
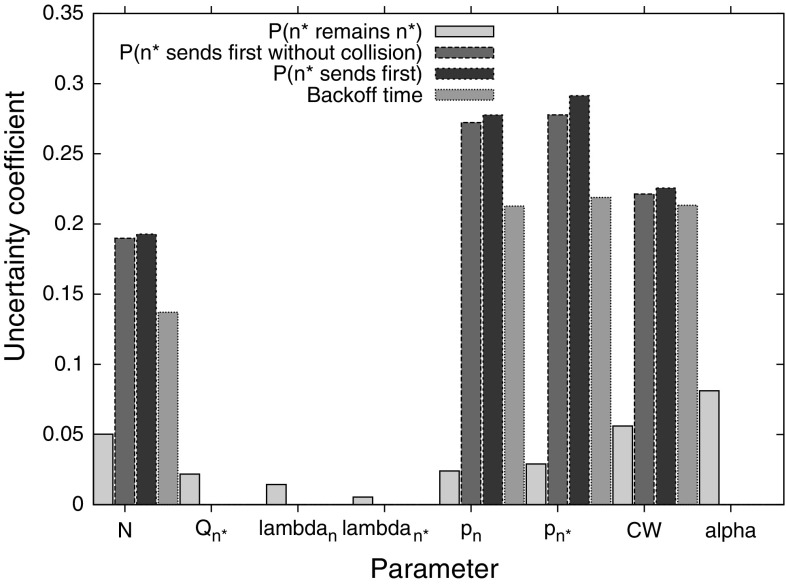
Uncertainty coefficients of each parameter to the model

Figure 2 shows the uncertainty coefficients for the input parameters for each model output. Both backoff time and $n^*$’s success in transmitting its packet first are governed by the countdown probabilities, contention window, and the number of nodes. In all cases, the number of nodes has the least impact. For $n^*$ to transmit its packet first, the most important parameters are the countdown probabilities, with $n^*$’s own countdown probability playing a particularly large role if $n^*$ should transmit its packet without collision. These results point to the importance of the function that selects countdown probabilities based on the nodes’ queue lengths and channel conditions, as the resulting performance is sensitive to the outputs of this function. Moreover, the operating conditions should be taken into consideration, with the function adjusted to suit the number of nodes in the network.

The probability that $n^*$ is still the highest-weighted node at the end of backoff can be seen to have the most complex relationship to the input parameters, with all parameters having some influence on the result. Note that since $Q_n$ is not varied in our dataset, its uncertainty coefficient is always 0 — it is the difference $Q_{n^*}-Q_n$, captured in our results simply by $Q_{n^*}$, that is important. We can see that $\alpha$ has a particularly large impact, indicating that bursty, high-variance traffic may adversely affect the performance of TO-DCF as the weighting of the nodes will change rapidly. This can result in a node that began with a lower weight actually having a higher weight at the end of backoff, due to the arrival of a burst of traffic, but not being the node that actually wins backoff, having been assigned a low countdown probability.

### Outputs

In the following sections, we will present results for each of the four model outputs. Here, in the interests of space, we have chosen to fix some parameters to representative values, and vary only those that our results from Sect. 6.2 indicate have a strong influence on each output. In all of the following figures, simulation results are shown with coloured lines, with 95% confidence intervals indicated, and the model results are overlaid as black crosses.

#### $P(n^*\, \mathrm {remains}\, n^*)$

**Fig. 3:**
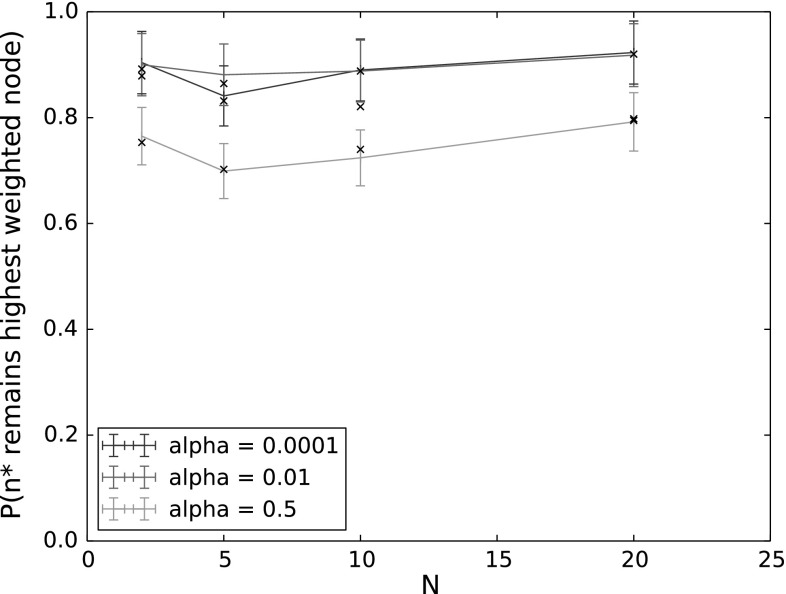
$P(n^*\, \mathrm {remains}\, n^*)$ for varying $\alpha$ and *N*. The other input parameters have the following values: $Q_{n^*}=2$, $\lambda _n=0.005$, $\lambda _{n^*}=0.001$, $p_{n^*}=p_n=0.9$, $CW=64$

Figure 3 shows the probability that $n^*$ is still the highest-weighted node at the end of backoff for varying number of nodes and $\alpha$. The other parameters were set to the following values: $Q_{n^*}=2$, $\lambda _n=0.005$, $\lambda _{n^*}=0.001$, $p_{n^*}=p_n=0.1$, $CW=64$. These parameter values were chosen so as to give the greatest disadvantage to $n^*$, that is, to make it as unlikely as possible that $n^*$ would indeed still be the node with the longest queue at the end of backoff.

**Fig. 4:**
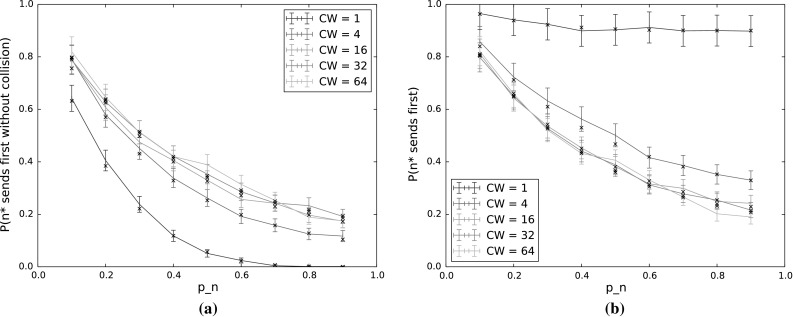
$P(n^*\,\mathrm {sends\,first\,without\,collision})$ and $P(n^*\,\mathrm {sends\,first})$ for varying $p_n$ and *CW*. The other input parameters have the following values: $Q_{n^*}=2$, $N=5$, $\lambda _n=\lambda _{n^*}=0.001$, $p_{n^*}=0.9$, $\alpha =0.5$. **a** $P(n^*\,\mathrm {sends\,first\,without\,collision})$. **b** $P(n^*\,\mathrm {sends\,first})$

As can be seen in the figure, even under these adverse conditions, the probability for $n^*$ to be the highest weighted node at the end of backoff nonetheless remains high in all cases. This indicates that for reasonable network loads. the changing weights of nodes due to packet arrivals during backoff is not an important consideration for the performance of the protocol and focus should instead be placed on minimising collision probabilities and idle time on the channel.

Our results also show a non-monotonic dependence on the number of nodes. This is because with few nodes, it is less likely that any other node will overtake $n^*$ to become the new highest-weighted node. However, fewer nodes also results in longer backoff times, particularly with low countdown probabilities as used here, giving more time for another node to potentially generate new packets and overtake $n^*$. Since $Q_{n^*}$ is here set to its lowest possible value of 2, only 2 packets need to arrive at another node (without $n^*$ generating any packets) for $n^*$ to no longer have the highest weight. These two conflicting effects can be seen in the figure, with the probability of $n^*$ remaining the highest-weighted node first decreasing, then increasing with increasing *N*.

#### $n^*$ transmits first

Figure 4 shows the probability that $n^*$ transmits first, without collision (Fig. 4a) and regardless of whether or not there is a collision (Fig. 4b), for varying values of $p_n$ and *CW*. Here, $p_{n^*}$ is held constant at 0.9. The difference between each pair of corresponding curves then gives the collision probability for that value of *CW*.

With increasing $p_n$, the collision probability increases and the probability that $n^*$ will transmit first decreases, even when we disregard collisions. Using TO-DCF, then, a low value of $p_n$ in comparison to $p_{n^*}$ thus gives better traffic separation, by giving priority to the node with the longest queue. The drop in $n^*$’s probability to transmit first is quite dramatic as $p_n$ increases, so it is important for $n^*$ to have a significant advantage in terms of countdown probability, especially for low contention window values. However, since nodes only have local information and do not have any indication of the average queue length across the network, this is easier said than done. It would therefore be of benefit to incorporate the anticipated or measured offered load in the network when selecting a weighting function to determine the countdown probabilities from the queue lengths.

#### Backoff time

**Fig. 5:**
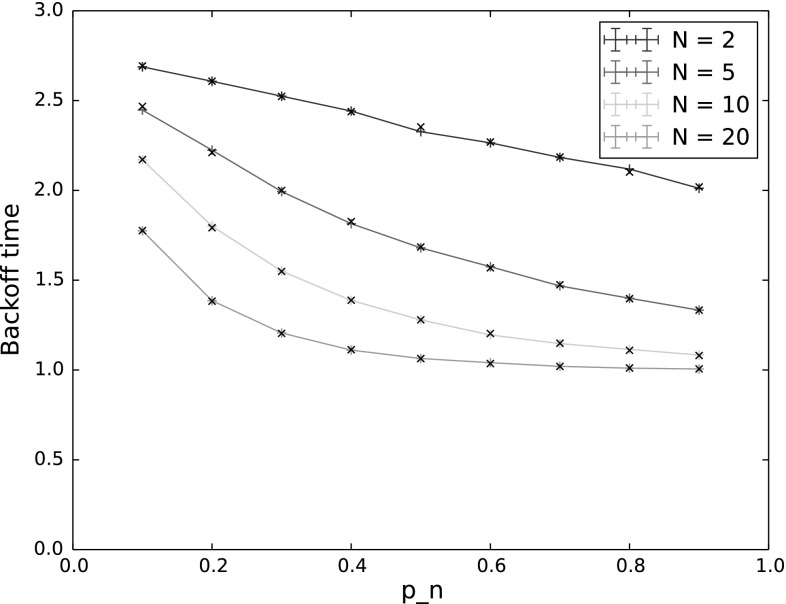
Backoff time for varying $p_n$ and *N*. The other input parameters have the following values: $Q_{n^*}=2$, $\lambda _n=\lambda _{n^*}=0.001$, $p_{n^*}=0.9$, $CW=4$, $\alpha =0.5$

Figure 5 shows the backoff time (mean for the simulations and expectation for the model), with varying $p_n$ and *N*. Again, $p_{n^*}$ is held constant at 0.9. Backoff time decreases with both increasing $p_n$ and increasing number of nodes. This is to be expected since as $p_n$ increases, the nodes decrement their backoff counters and reach 0 faster, and when there are more nodes, the chances of at least one node counting down to 0 within a given time increase.

**Fig. 6:**
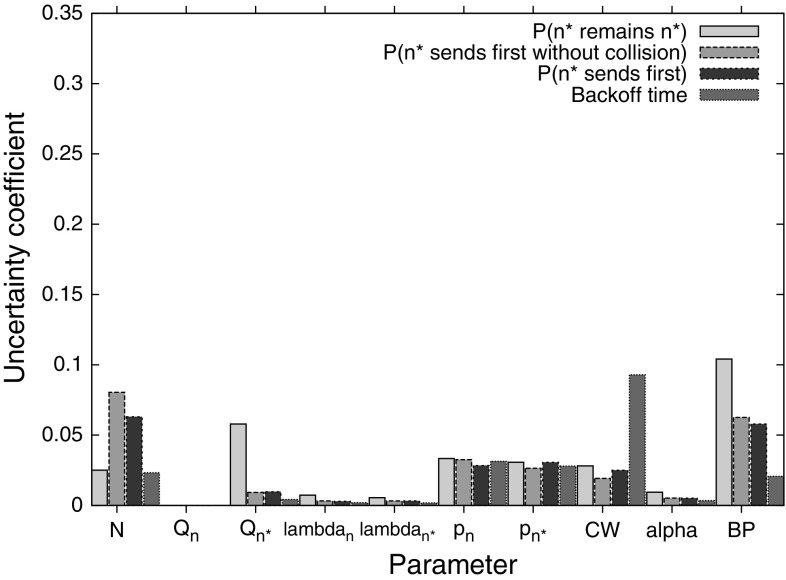
Uncertainty coefficients for simulations with multiple transmissions

**Fig. 7:**
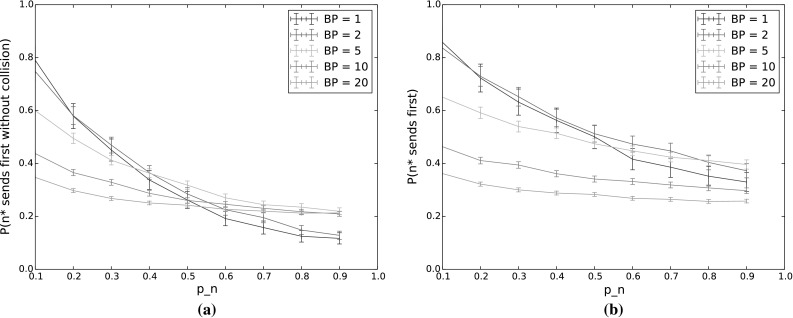
$P(n^*\,\mathrm {sends\,first\,without\,collision})$ and $P(n^*\,\mathrm {sends\,first})$ for varying $p_n$ as the number of backoff periods (BP) increases. The other input parameters have the following values: $Q_{n^*}=2$, $N=5$, $\lambda _n=\lambda _{n^*}=0.001$, $p_{n^*}=0.9$, $CW=4$, $\alpha =0.5$. **a** $P(n^*\,\mathrm {sends\,first\,without\,collision})$. **b** $P(n^*\,\mathrm {sends\,first})$

In terms of the performance of the protocol, backoff time gives an indication of both delay and throughput. Time spent in backoff not only delays the actual transmission of packets, but is also idle time for the entire network, wasting capacity on the channel. Thus to achieve good performance, backoff time should be reduced. However, this must be weighed against the goals of the protocol. As we have seen in Sect. 6.3.2, the probability of $n^*$ sending first decreases with increased $p_n$, and the probability of a collision increases, especially for low values of *CW*. We can therefore see that setting the countdown probabilities for the number of nodes and contention window size is critical to achieving both good performance and traffic separation with TO-DCF, and our model provides a means of calculating appropriate values.

### Multiple transmissions

We now investigate the effects of our model assumptions, given in Sect. 3.2, by conducting simulations in which these assumptions are relaxed. First we tested the operation of TO-DCF over multiple transmissions, that is, multiple backoff periods. This removes the assumptions that nodes will all begin backoff at the same time and that all nodes participate in backoff; the number of nodes participating in backoff will change as nodes transmit their packets and no longer have any packets remaining in their queues.

Figure 6 shows the uncertainty coefficients for the various input parameters, as well as a new parameter: the number of backoff periods, labelled “BP” in the figure. Since our model does not cover multiple transmissions, here the uncertainty coefficients are calculated relative to the simulation results, rather than the model results as was done in Sect. 6.2. This means that the overall variation in the results is increased due to the random number generation involved in the simulations, and thus all uncertainty coefficients are lower. However, the relative values of the coefficients can nonetheless give insight into how the effects of the input parameters are changed when we move to a situation with multiple transmissions.

Overall the patterns of uncertainty coefficients are similar to the single backoff period case, across all the outputs. The biggest difference is that now the number of backoff periods has a sizeable effect on all of the outputs with the exception of backoff time. However, as our results in the following sections illustrate, this is largely due to the changing number of nodes. When multiple transmission are allowed, nodes that empty their queues no longer participate in backoff, affecting the output values. To investigate this, we tested a saturated network, that is, a network in which all nodes always have a packet in their queue to send.

**Fig. 8:**
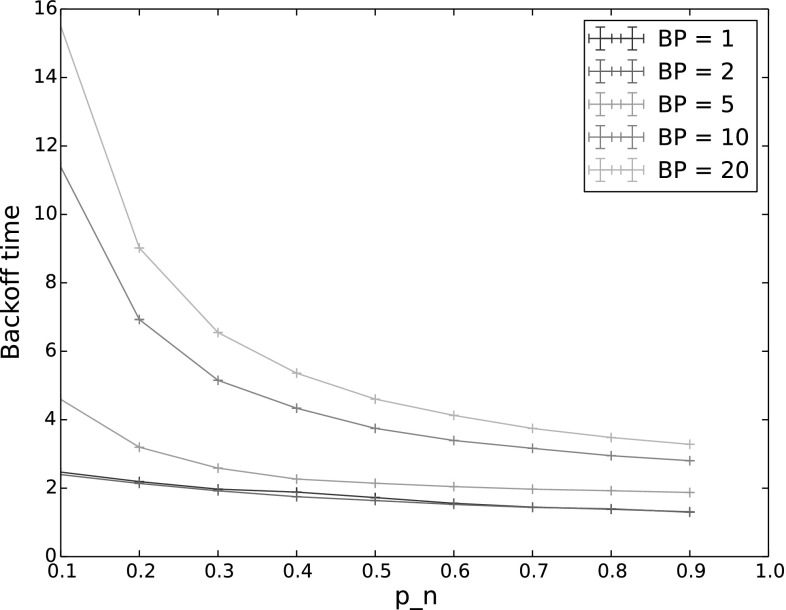
Backoff time for varying $p_n$ as the number of backoff periods (BP) increases. The other input parameters have the following values: $Q_{n^*}=2$, $N=5$, $\lambda _n=\lambda _{n^*}=0.001$, $p_{n^*}=0.9$, $CW=4$, $\alpha =0.5$

**Fig. 9:**
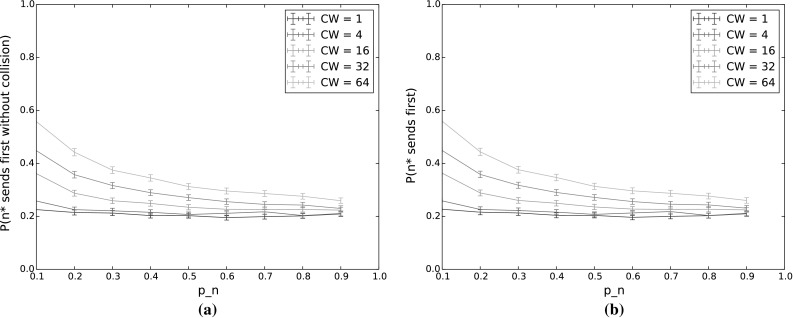
$P(n^*\,\mathrm {sends\,first\,without\,collision})$ and $P(n^*\,\mathrm {sends\,first})$ for varying $p_n$ and *CW*, in steady state ($\hbox {number of transmissions} = 1000$). The other input parameters have the following values: $Q_{n^*}=2$, $N=5$, $\lambda _n=\lambda _{n^*}=0.001$, $p_{n^*}=0.9$, $CW=4$, $\alpha =0.5$. **a** $P(n^*\,\mathrm {sends\,first\,without\,collision})$. **b** $P(n^*\,\mathrm {sends\,first})$

As we increase the number of backoff periods, we see a fairly smooth transition from the results given above for a single backoff period, to the steady state results shown in the next section. In all cases, including steady state, the probability for $n^*$ to still be the highest-weighted node at the end of backoff remained high, with similar results to those shown in Fig. 3. We therefore omit results for this output in the following and show only results for the probability for $n^*$ to transmit first, with and without collisions (Fig. 7) and backoff time (Fig. 8). While the backoff time varies monotonically with the number of backoff periods, this is not true for the probability that $n^*$ transmits first. This is because, as mentioned previously, fewer nodes actually participate in later backoff periods due to not having a packet to send in their queues. This means that the probability of collision first decreases, primarily affecting high values of $p_n$, and then the probability of sending first slowly transitions to a base probability of which node (out of the 5 in the experiment) actually receives a packet to send. These effects are discussed in more detail in the following section.

**Fig. 10:**
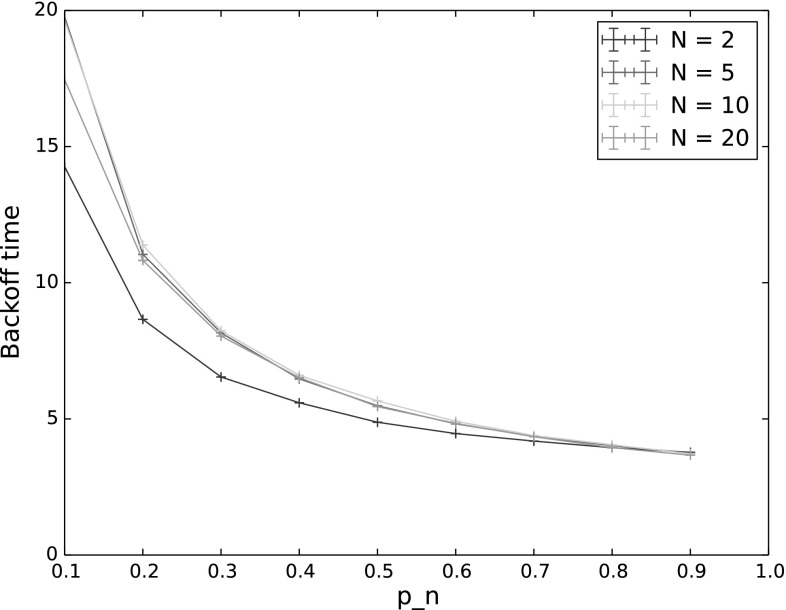
Backoff time for varying $p_n$, in steady state ($\hbox {number of transmissions} = 1000$). The other input parameters have the following values: $Q_{n^*}=2$, $\lambda _n=\lambda _{n^*}=0.001$, $p_{n^*}=0.9$, $CW=4$,$\alpha =0.5$

#### Steady state

To obtain results for the system in steady state, we ran simulations for 1000 backoff periods. It is possible that we do not thus obtain a true steady state, that is, that the system has reached stationary probabilities, however our simulation results indicate stable behaviour at this large timescale. The probability for $n^*$ to transmit first, with and without collisions, is shown in Fig. 9. We can see from the similarity of the two cases (with and without collisions) that the collision probability is very low. Further, the overall probability for $n^*$ to transmit first is much lower than in the single backoff period case.

**Fig. 11:**
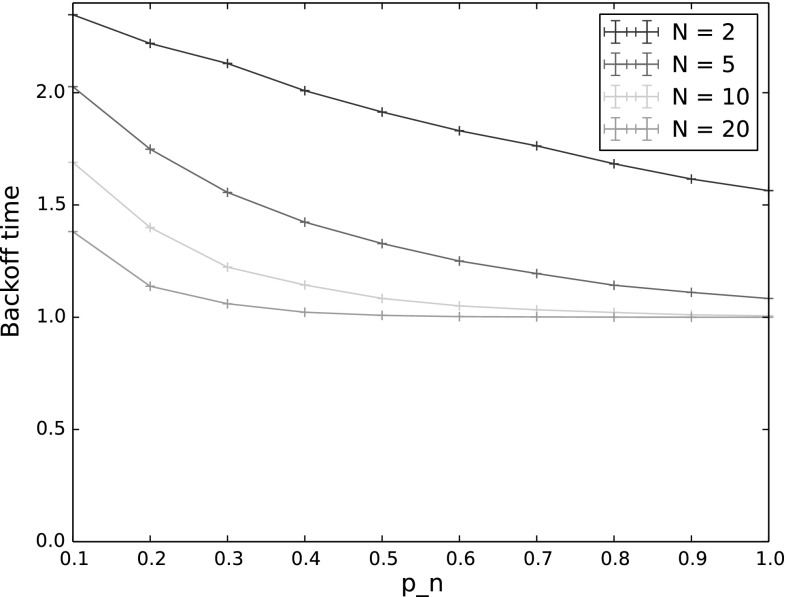
Backoff time for varying $p_n$ and *N*, in steady state ($\hbox {number of transmissions} = 1000$), for a saturated network. The other input parameters have the following values: $Q_{n^*}=2$, $\lambda _n=\lambda _{n^*}=0.001$, $p_{n^*}=0.9$, $CW=4$,$\alpha =0.5$

**Fig. 12:**
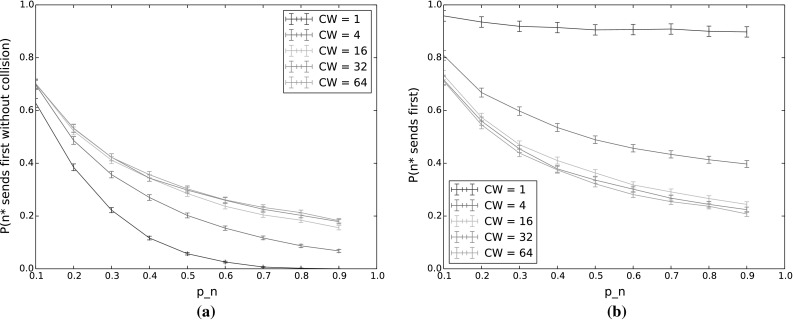
$P(n^*\,\mathrm {sends\,first\,without\,collision})$ and $P(n^*\,\mathrm {sends\,first})$ for varying $p_n$ and *CW*, in steady state ($\hbox {number of transmissions} = 1000$), for a saturated network. The other input parameters have the following values: $Q_{n^*}=2$, $N=5$, $\lambda _n=\lambda _{n^*}=0.001$, $p_{n^*}=0.9$, $\alpha =0.5$. **a** $P(n^*\,\mathrm {sends\,first\,without\,collision})$. **b** $P(n^*\,\mathrm {sends\,first})$

This can be explained by the effect of nodes dropping out of the backoff process as they transmit their packets and empty their queues. Because our values of $\lambda$ are relatively low in order to ensure the network is not overloaded, in the steady state, each node spends a significant amount of its time not participating in backoff as it is waiting for a packet to arrive in its erstwhile empty queue. However, the node designated as $n^*$ is chosen only when a transmission occurs, and if all nodes have equal length queues, the designation of $n^*$ is arbitrary. Hence if all nodes have empty queues after a transmission, the node designated as $n^*$ may not be the node that receives the most packets by the time the next backoff period actually starts.

This effect can also be seen in Fig. 10, which shows backoff time in steady state. Here we see that increasing the number of nodes does not significantly alter the backoff time except for very low values of $p_n$, in which case backoff times are longer and nodes are thus less likely to haove empty queues at the end of backoff.

To eliminate the effect of nodes dropping out of the backoff process, we tested a case in which the network was saturated. Here, each node began with 1000 packets in its queue (except for $n^*$ which began with 1001 packets). This means that the nodes are not able to empty their queues over 1000 backoff periods and thus all nodes will participate in backoff for the entire duration of the simulation. The results for the saturated network are shown in Figs. 11 and 12.

For the saturated case, we see that even in steady state, that is, with a large number of backoff periods, the results match the model results quite closely. We can therefore conclude that the assumption of a single backoff period does not have a significant effect on the accuracy of the model results, however, nodes dropping out of backoff due to emptying their queues does indeed have a large effect. This means that in order to use the model to calculate operating parameters for a real network, the average number of nodes actually participating in backoff (based on the load of the network) should be used, rather than the overall number of nodes in the network.

#### Exponential backoff

**Fig. 13:**
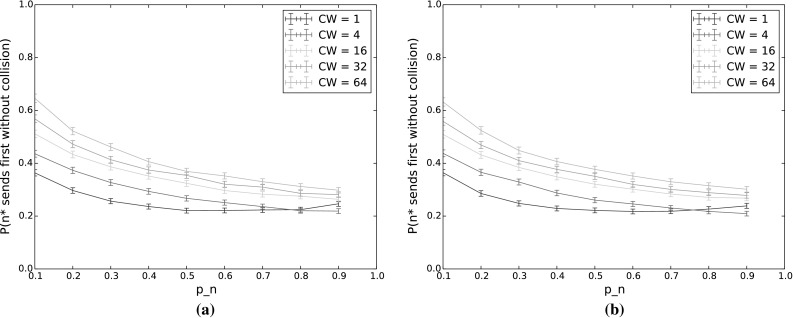
$P(n^*\,\mathrm {sends\,first\,without\,collision})$ with and without exponential backoff, for 10 transmissions. The other input parameters have the following values: $Q_{n^*}=2$, $N=5$, $\lambda _n=\lambda _{n^*}=0.001$, $p_{n^*}=0.9$, $\alpha =0.5$. **a** Exponential backoff. **b** No exponential backoff

**Fig. 14:**
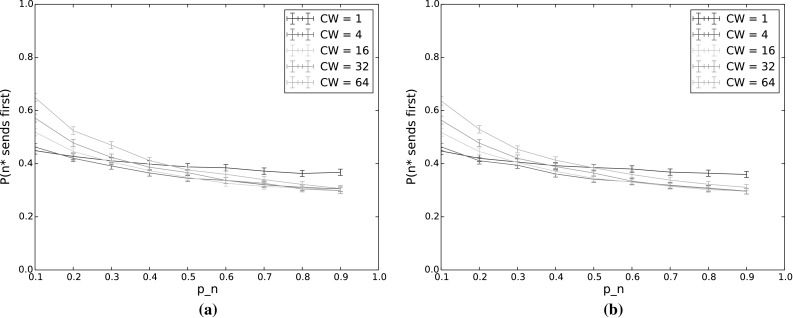
$P(n^*\,\mathrm {sends\,first})$ (regardless of collisions), with and without exponential backoff, for 10 transmissions. The other input parameters have the following values: $Q_{n^*}=2$, $N=5$, $\lambda _n=\lambda _{n^*}=0.001$, $p_{n^*}=0.9$, $\alpha =0.5$. **a** Exponential backoff. **b** No exponential backoff

**Fig. 15:**
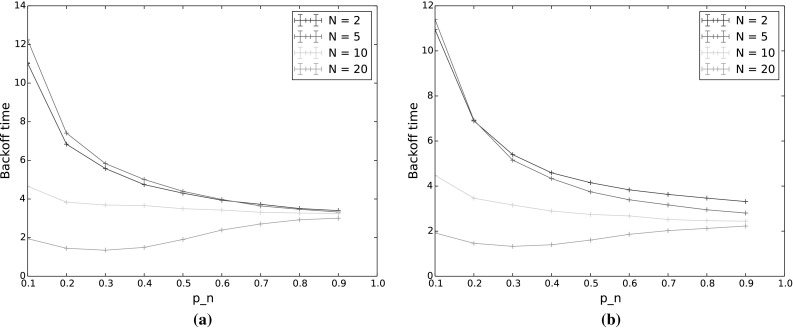
Backoff time with and without exponential backoff, for 10 transmissions. The other input parameters have the following values: $Q_{n^*}=2$, $\lambda _n=\lambda _{n^*}=0.001$, $p_{n^*}=0.9$, $CW=4$,$\alpha =0.5$. **a** Exponential backoff. **b** No exponential backoff

Next, we tested the effect of exponential backoff. Side by side results for simulations over 10 backoff periods, with and without exponential backoff, are shown in Figs. 13, 14, and 15. From the figures, we can see that exponential backoff has very little effect on the probability for $n^*$ to transmit first, whether or not we include collisions. Using exponential backoff does have some effect on the backoff time. Backoff times are higher under exponential backoff for a large number of nodes and high $p_n$, and for a small number of nodes regardless of $p_n$. In the case of high $p_n$, collisions are more likely so exponential backoff has a larger effect. In the case of a low number of nodes, the effect of individual nodes entering exponential backoff is larger since there are so few nodes in the system and thus again, exponential backoff has a larger effect. However, in all cases the difference in outputs with and without exponential backoff is small.

#### Dense networks

**Fig. 16:**
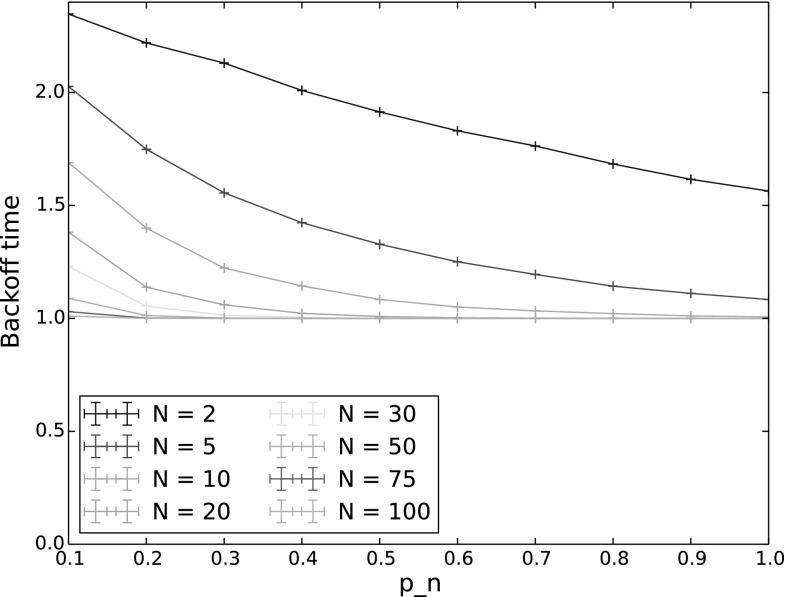
Backoff time in steady state for varying $p_n$ and number of nodes, saturated network. The other input parameters have the following values: $Q_n = 1000$, $Q_{n^*}=1001$, $\lambda _n=\lambda _{n^*}=0.001$, $p_{n^*}=0.9$, $CW=4$, $\alpha =0.5$

For our final set of experiments, we investigated how increasing network density, i.e. a larger number of nodes, affects backoff time. Here, we conducted simulations in steady state (1000 backoff periods), with the number of nodes increasing from 2 to 100, for both a saturated network (all nodes begin with at least 1000 packets in their queues), and a non-saturated network. The results of these experiments are shown in Figs. 16 (saturated network) and 17 (non-saturated network). Comparing a saturated and non-saturated network allows us to see the effect on steady state backoff time of the effective number of nodes reducing over time due to not all nodes having packets to send. As would be expected, the backoff time is much longer in a non-saturated network (note the different *y*-scales in the two figures). While the number of nodes does clearly play a role in determining backoff time, the arrival rate of packets for the network as a whole has a bigger effect than how many nodes those packets are distributed amongst.

**Fig. 17:**
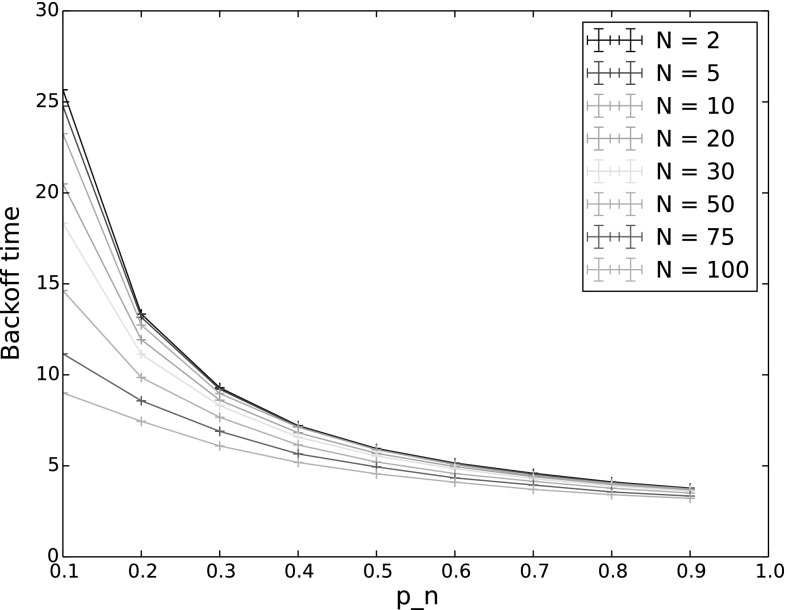
Backoff time in steady state for varying $p_n$ and number of nodes, non-saturated network. The other input parameters have the following values: $Q_{n^*}=2$, $\lambda _n=\lambda _{n^*}=0.001$, $p_{n^*}=0.9$, $CW=4$, $\alpha =0.5$

## Conclusion

In this work we have provided an in-depth analysis of the TO-DCF backoff scheme and numerically evaluated the expressions found, as well as conducted simulations, in order to provide insight into the performance and behaviour of this scheme. We determined the expected backoff time, along with the probabilities associated with various success metrics for TO-DCF. Our numerical and simulation results demonstrate the accuracy of our model, as well as illustrating that the assumptions used in creating the model have minimal effect on the correctness of the results. The one exception to this was the assumption of all nodes in the network participating in the backoff process. The number of nodes was found to have a profound effect and as such it is important to use the actual number of nodes participating in backoff as input to the model to ensure reliable results. We also examined the steady-state behaviour of the system and found it matched our model results well in the case of saturation, where all nodes participate in backoff.

Our calculations of the uncertainty coefficients of the input parameters to the model show that the countdown probabilities, and in particular their spread, are especially important in determining the performance of TO-DCF. These values strongly affect backoff time, collision probability and the likelihood of the highest weighted node winning contention and sending its packet first, thus achieving the desired traffic separation. On the other hand, packet arrivals, even with high variance, did not have a large effect on the performance for a network with achievable load.

When applying protocols in real-world deployments, it is essential to have a thorough understanding of the behaviour of these systems and how performance will be affected by operating conditions and input parameter values. Trade-offs such as those we have seen in this work arise naturally in such systems where behaviour is governed by stochastic processes and performance is defined in terms of multiple, often conflicting, goals. TO-DCF is a promising scheme that has the potential to dramatically improve performance in wireless LANs, and our analysis and numerical results provide network operators with the understanding required to effectively deploy this scheme, making it viable as a solution to the increasing demands of future networks.
